# The antitumour activity of 2‐(4‐amino‐3‐methylphenyl)‐5‐fluorobenzothiazole in human gastric cancer models is mediated by AhR signalling

**DOI:** 10.1111/jcmm.14869

**Published:** 2019-12-25

**Authors:** Yuling Wang, Ying Liu, Tao Tang, Ying Luo, Malcolm F. G. Stevens, Xi Cheng, Yan Yang, Dongfang Shi, Jihong Zhang, Tracey D. Bradshaw

**Affiliations:** ^1^ Laboratory of Molecular Genetics of Aging and Tumor Medical School Kunming University of Science and Technology Kunming China; ^2^ School of Pharmacy Centre for Biomolecular Sciences University of Nottingham Nottingham UK; ^3^ Atom Bioscience and Pharmaceutical Co., Ltd. Zhenjiang China

**Keywords:** arylhydrocarbon (AhR) receptor, CYP‐catalysed bioactivation, molecular pharmacodynamic (PD) markers, stomach cancer

## Abstract

Stomach cancer is the fourth most common cancer worldwide. Identification of novel molecular therapeutic targets and development of novel treatments are critical. Against a panel of gastric carcinoma cell lines, the activity of 2‐(4‐amino‐3‐methylphenyl)‐5‐fluorobenzothiazole (5F 203) was investigated. Adopting RT‐PCR, Western blot and immunohistochemical techniques, we sought to determine molecular pharmacodynamic (PD) markers of sensitivity and investigate arylhydrocarbon (AhR) receptor‐mediated signal transduction activation by 5F 203. Potent (IC_50_ ≤ 0.09 μmol/L), selective (>250‐fold) in vitro antitumour activity was observed in MKN‐45 and AGS carcinoma cells. Exposure of MKN‐45 cells to 5F 203 triggered cytosolic AhR translocation to nuclei, inducing CYP1A1 (>50‐fold) and CYP2W1 (~20‐fold) transcription and protein (CYP1A1 and CYP2W1) expression. G2/M arrest and γH2AX expression preceded apoptosis, evidenced by PARP cleavage. In vivo, significant (*P *< .01) 5F 203 efficacy was observed against MKN‐45 and AGS xenografts. In mice‐bearing 5F 203‐sensitive MKN‐45 and 5F 203‐insensitive BGC‐823 tumours in opposite flanks, CYP1A1, CYP2W1 and γH2AX protein in MKN‐45 tumours only following treatment of mice with 5F 203 (5 mg/kg) revealed PD biomarkers of sensitivity. 5F 203 evokes potent, selective antitumour activity in vitro and in vivo in human gastric cancer models. It triggers AhR signal transduction, CYP‐catalysed bioactivation to electrophilic species causing lethal DNA double‐strand breaks exclusively in sensitive cells. 5F 203 represents a novel therapeutic agent with a mechanism of action distinct from current clinical drugs, exploiting novel molecular targets pertinent to gastric tumourigenesis: AhR, CYP1A1 and CYP2W1. PD markers of 5F 203 sensitivity that could guide patient selection have been identified.

## 1 INTRODUCTION

Stomach cancer is the fourth most common cancer and second leading cause of cancer death worldwide. With >950 000 new diagnoses each year and ~720 000 deaths in 2012, gastric cancer represents 7% of global cancer incidence.^1^ Although worldwide, the incidence of gastric cancer is declining, it remains highly prevalent in Asia when compared to the West. China is one of the countries with the highest incidence of gastric cancer and accounts for >40% of all new gastric cancer cases in the world. Indeed, gastric cancer is the third leading cause of cancer mortality in China. Globally, 5‐year survival for gastric cancer is ~30%; for localized disease, 67% of patients survive beyond 5 years, but >70% gastric cancer patients possess metastases (in regional lymph nodes or at distant sites) at the time of diagnosis. The prognosis for patients with metastatic disease is very poor: median survival is between 4 months (with supportive care) and 12 months (with combination cytotoxic chemotherapy); 5‐year survival statistics are dismal (~5%).

Treatment of this malignancy includes surgery, radiation therapy and chemotherapy, but clinical practice varies widely internationally. Most chemotherapy treatments for stomach cancer are based on the combination of at least 2 drugs, 5‐fluorouracil and cisplatin, or related derivatives capecitabine, and oxaliplatin. Other drugs commonly used include paclitaxel, docetaxel, epirubicin and irinotecan. Systemic toxicities associated with such cytotoxic chemotherapy regimens include myelosuppression, heightened infection risk, fatigue, nausea and vomiting, hair loss, loss of appetite and diarrhoea and are often dose‐limiting.^2^ Although targeted therapies, for example trastuzumab (anti‐HER2 antibody) and ramucirumab (anti‐VEGFR2 antibody), have recently been introduced clinically, currently, there remains no internationally accepted standard treatment regimen for this intractable disease. Thus, gastric cancer presents a significant health burden and unmet clinical need, and development of novel treatments for this insidious disease is essential to improve the outcomes and quality of life for gastric cancer patients.

**Figure 1:**
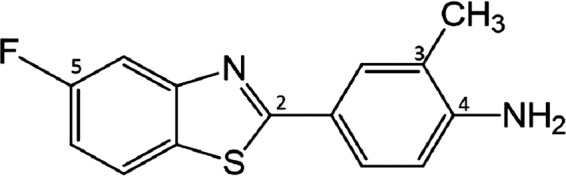
Structure of 2‐(4‐amino‐3‐methylphenyl)‐5‐fluorobenzothiazole (5F 203)

The benzothiazole pharmacophore has proved important in medicinal chemistry: agents built upon this scaffold possess activity in multiple medicinal and clinical fields—including cancer. 2‐(4‐Amino‐3‐methylphenyl)‐5‐fluorobenzothiazole (5F 203; Figure 1) elicits potent, selective antitumour activity in vitro and in vivo^3^ via a unique mechanism of action. Potent arylhydrocarbon receptor (AhR) ligands,^4^ benzothiazoles induce their own cytochrome P450‐mediated (CYP 1A1; CYP 2W1) biotransformation to electrophilic nitrenium species in sensitive tumour cells only.^5^, ^6^ These reactive species generate DNA adducts (N7‐guanine), which lead to lethal double DNA strand breaks^7^, ^8^—exclusively in cancer cells expressing cytosolic AhR and inducible CYP1A1 or CYP2W1. Correlation has been shown between inducible CYP1A1 and sensitivity to 5F 203 in the NCI 60 cell line panel. Sensitivity to 5F 203 in vitro, in vivo and ex vivo in human breast and ovarian tumour models correlated with induction of CYP1A1.^9^, ^10^ Indeed, following ex vivo exposure of human tumour cells isolated from ovarian carcinoma patients, induction of CYP1A1 and nuclear translocation of AhR, respectively, have been shown to correlate with sensitivity to 5F 203.^9^, ^11^ Expression of CYP2W1, normally restricted to the embryonic period, re‐emerges in certain colorectal and hepatocellular carcinomas portending progressive disease and poor prognosis.^12^, ^13^ CYP2W1 expression in colorectal carcinoma cells renders them sensitive to antitumour benzothiazoles such as 5F 203 and GW 610, catalysing their conversion to reactive species.^14^ 5F 203 lysylamide prodrug (Phortress)^10^, ^15^, ^16^ underwent phase 1 clinical evaluation and was generally well tolerated. Stable disease was achieved in 28% patients receiving Phortress, with long‐term stable disease observed in 4 (2 renal, 1 colon and 1 mesothelioma) patients, neither breast nor ovarian carcinoma patients being recruited to the trial.

To date, the effect of antitumour benzothiazoles on gastric cancer models has not been investigated. Given that aberrant AhR expression and pathway activation are involved in gastric carcinogenesis;^17^ both CYP1A1 mRNA and protein expression are inducible in gastric cancer; CYP2W1 protein is embryonically expressed in gastrointestinal tissues and highly expressed in gastric cancer;^18^ we speculate that AhR, CYP1A1 and tumour‐specific CYP2W1 may represent putative molecular targets for anticancer therapy in gastric cancer. We thus tested the hypothesis that certain gastric cancer models demonstrate sensitivity to the antitumour benzothiazole 5F 203.

Herein, we report selective and potent activity of 5F 203 in certain gastric carcinoma models in vitro (nM sensitivity) and in vivo. Cytosolic AhR expression was demonstrated in MKN‐45 cells; sensitivity to 5F 203 (a potent AhR ligand) correlated with CYP1A1 and CYP2W1 mRNA and protein induction. 5F 203 evoked S‐ and G2‐phase cell cycle arrest (at 24 hours and 48 hours, respectively), generation of DNA double‐strand breaks as evidenced by γH2AX detection (≥6 hours exposure) and subsequent PARP cleavage signalling cell death (≥48 hours). In vivo 5F 203 efficacy was observed against MKN‐45 and AGS xenografts, and selective induction of CYP1A1, CYP2W1 and γH2AX protein expression, detected in MKN‐45, but not 5F 203‐insensitive BGC‐823 tumours excised 24 hours post‐treatment of mice (5 mg/kg 5F 203) indicate AhR signal cascade activation in vivo.

We conclude that stomach cancers whose tumour cells express cytosolic AhR are likely to be sensitive to 5F 203 and that 5F 203 represents a putative novel therapeutic agent in treatment of certain gastric cancers.

## 2 MATERIALS AND METHODS

### 2.1 Chemicals

5F 203 was synthesized at the University of Nottingham (UK), prepared as 10 mmol/L stock solutions in dimethyl sulfoxide (DMSO), stored in aliquots and protected from light at –20°C. Reagents, unless specified otherwise, originated from Sigma‐Aldrich Ltd. Materials used and methods conducted at Atom Bioscience and Pharmaceutical Co. Ltd. are described in Supplementary Information (SI).

### 2.2 Cell lines and culture conditions

Gastric cancer cell lines MKN‐45, KATO III, NCI‐N87, SGC‐7901 and BGC‐823, were obtained from the Chinese Academy of Sciences cell bank. Cells were cultured in RPMI 1640 medium supplemented with 10% foetal bovine serum (FBS). MCF‐7 HCT116, HepG2 and PC‐3 cells, obtained from the American Type Culture Collection (ATCC), were grown in RPMI 1640 medium supplemented with 10% FBS. Cells were incubated in a humidified atmosphere of 95% air and 5% CO_2_ at 37°C.

### 2.3 MTT assay

Cells were seeded into 96‐well plates at a density of 4 × 10^3^ per well and allowed to attach overnight. Dilutions of 5F 203 (0.01‐50 μmol/L) were prepared in culture medium from 10 mmol/L stock solutions and following 3 days of incubation (37°C, 5% CO_2_). Sterile‐filtered 3‐(4,5‐dimethylthiazol‐2‐yl)‐2,5‐diphenyltetrazolium bromide (MTT) (20 μL; 5 mg/mL in phosphate buffered saline) was added to each well (final concentration 0.4 mg/mL). Plates were re‐incubated for 4 hours allowing metabolism of MTT by viable cells to insoluble formazan crystals. Medium and unconverted MTT were aspirated, and DMSO (150 μL) was added to each well to ensure complete formazan solubilization; absorbance was read on a BioTek SynergyH1 microplate reader (490 nm). Compound concentrations causing 50% inhibition (IC_50_) values were calculated by interpolation.

### 2.4 Western blot analysis

Cells were lysed in RIPA lysis buffer (25 mmol/L Tris HCl (pH 7.5), 2.5 mmol/L EDTA, 2.5 mmol/L EGTA, 20 mmol/L NaF, 1 mmol/L Na_3_VO_4_, 100 mmol/L NaCl, 20 mmol/L sodium ‐glycerophosphate, 10 mmol/L sodium pyrophosphate and 0.5% triton X‐100) supplemented with a protease inhibitor cocktail (Roche). Cellular proteins (30 μg) were separated by SDS‐PAGE and electro‐transferred onto PVDF membranes. Membranes were blocked in Tris‐buffered saline (TBS) containing 5% milk and 0.1% Tween‐20 at room temperature. Membrane incubation with 1° Abs (AhR, CYP1A1, PCNA, γH2AX, PARP and beta‐actin, sourced from Santa Cruz or Cell Signaling) was conducted overnight at 4°C. Membranes were washed at room temperature before incubation with 2° Ab (GE) conjugated with horseradish peroxidase for 1 hour. Detection was performed with Super Signal Chemiluminescent reagent according to the manufacturer's protocol (Tanon, China).

### 2.5 Cell cycle analysis

Exponentially growing cells were harvested and seeded in 6‐well plates (2 × 10^5^ cells/well; 2 mL medium). Cells were incubated overnight and then treated with 1 μmol/L 5F 203 for 3, 6, 12, 24 or 48 hours. Attached and floating cells were pooled and pelleted by centrifugation (132*g*, at 4°C, 5 minutes), and cell pellets were washed with PBS and re‐suspended in 0.3 mL hypotonic fluorochrome solution (0.1% sodium citrate, 0.1% Triton X‐100, 50 g/mL PI and 0.1 mg/mL ribonuclease A) and then stored overnight at 4°C in the dark. Fluorescence of PI‐stained DNA was detected on a BD C6 cytometer, and data were analysed using C6 software.

### 2.6 RT‐PCR

Human gastric cancer MKN‐45 cells were treated with 1 μmol/L 5F 203 for 3, 6 and 12 hours. Total RNA was extracted by using TRIzol and incubated with RNase‐free DNase. Primers for CYP1A1, CYP1B1, CYP2S1 and CYP2W1 were designed with Primer Express software (Applied Biosystems) using the gene bank sequence for human CYP1A1 mRNA (forward 5′‐TCGGCCACGGAGTTTCTTC‐3′, reverse 5′‐GGTCAGCATGTGCCCAATCA‐3′), human CYP1B1 mRNA (forward 5′‐TGAGTGCCGTGTGTTTCGG‐3′, reverse 5′‐GTTGCTGAAGTTGCGGTTGAG‐3′), human CYP2S1 mRNA (forward 5′‐GCGCTGTATTCAGGGCTCAT‐3′, reverse 5′‐CTTCCAGCATCGCTACGGTT‐3′, human CYP2W1 mRNA (forward 5′‐AGCTATGTGGACGCCCTGATCCA‐3′, reverse 5′‐ACGCGTCTAGCTCCTCCTGCAC‐3′). cDNAs were synthesized from RNA (2 µg) using promega GoScript cDNA synthesis kit according to manufacturer's instruction. SYBR Green PCR amplification was performed with real‐time PCR system.

### 2.7 Nuclear and cytoplasmic protein extraction

Nuclear and cytoplasmic protein extraction was performed with the Nuclear and Cytoplasmic Protein Extraction Kit (Beyotime P0028) in accordance with manufacturer's instructions. MNK‐45 cells were treated with 1 μmol/L 5F 203 for 1, 2, 3, 6, 24, 48 and 72 hours, and treated cells were collected and lysed with 200 µL cytoplasmic protein extraction reagent A (adding PMSF; 1 mmol/L final concentration). Samples were re‐suspended with a pipette and placed on ice for 15 minutes before addition of 10 µL cytoplasmic protein extraction reagent B. Samples were subjected to vigorous vortexing for 5 seconds and then placed on ice. After further centrifugation (16 000 *g*; 4°C; 5 minutes), samples were lysed with 50 µL Nuclear Protein Extraction Reagent (adding PMSF to yield 1 mmol/L final concentration) and mixed vigorously with a pipette. The mixture was then repeatedly vortexed for 30 seconds and placed on ice for 2 minutes. Finally, samples were centrifuged (16 000 *g*; 4°C) for 15 minutes.

### 2.8 Immunofluorescence

Cells were fixed with fixative solution (3% paraformaldehyde and 2% sucrose solution) and permeabilized with 1% NP‐40. Cells were blocked with 5% bovine serum albumin (BSA) for 2 hours before incubation with Abs. AhR (H‐211, 1:50 dilution, Santa Cruz) and CYP2W1 (c‐7, 1:50 dilution, Santa Cruz) Abs were incubated at 4°C overnight. 2° Abs Alexa Fluor 488 goat anti‐Rabbit IgG (A11034, 1:1500, Invitrogen) and Alexa Fluor 488 goat anti‐Mouse IgG (A11001, 1:1500, Invitrogen). Samples were stained (DAPI; Invitrogen) for 20 minutes and mounted in the Antifade Mounting Medium, followed by analysis with a Nikon epifluorescence microscope.

### 2.9 In vivo studies

Balb/c nude mice (6‐8 weeks old, 18‐20 g) were purchased from Hunan SJA Laboratory Animal Co., Ltd. BGC‐823 and MKN‐45 (2 × 10^6^) cells were subcutaneously (SC) injected into the left and right flanks, respectively, of nude mice. When the average tumour volume reached 100 mm^3^, mice were randomly divided into 3 groups and administered ip with a vehicle of saline/Tween 80, 5 mg/kg and 10 mg/kg 5F 203 for consecutive days (days 0‐3). For tumour tissue analysis, nude mice‐bearing s.c. MKN‐45 cells were treated with 5 mg/kg 5F 203 for 24 hours and tumour tissues were retrieved, fixed with 10% formaldehyde or snap frozen.

Tissue samples were lysed in RIPA buffer containing Protease Inhibitor Cocktail kit (Roche). Total proteins (50 µg) were separated by SDS‐PAGE and then transferred to PVDF membranes. After blocking in 2% BSA for 1 hour at room temperature, membranes were incubated with 1° Abs overnight at 4°C or 2 hours at room temperature. The membranes were then incubated with horseradish peroxidase‐labelled 2° Abs and visualized with ECL. For tissue quantitative real‐time PCR analysis: RNA was isolated from tissue samples, and cDNA was synthesized by reverse transcription. Real‐time PCR was performed on an ABI Prism 7300 sequence detection system with SYBR Green PCR master mix following the manufacturer's instruction (Applied Biosystems, CA). Immunohistochemical analysis was performed as described previously.^19^ Briefly, slides from formalin‐fixed paraffin‐embedded tissue blocks were deparaffinised and endogenous peroxidase activity was inhibited using H_2_O_2_. Samples were then stained using 1° Abs (1:50) at 4°C overnight. Goat anti‐rabbit or mouse IgG/horseradish peroxidase was applied as 2° Abs according to the standard protocols provided by the manufacturer, followed by incubation of Vectastain ABC Kit (Vector Laboratories). Slides were examined under an inverted microscope at 200× magnification (Eclipse TS100, Nikon).

## 3 RESULTS

### 3.1 Activity of 5F 203 against gastric cancer cell lines

The activity of 5F 203 was tested against gastric cancer cell lines, 5F 203 demonstrated potency against MKN‐45 and AGS cells with mean IC_50_ values of ≤0.09 μmol/L and ~0.04 μmol/L, respectively, and the activity is similar to that observed in MCF‐7 cells which are already known to be sensitive to 5F 203.^3^ However, 5F 203 is inactive against SGC‐7901, BGC‐823 and KATO III gastric cancer cells (IC_50_ values >50 μmol/L; Table 1). Kunming NCI‐N87 cells demonstrated resistance to 5F 203 (mean IC_50_ value 22.63 μmol/L), and however, against NCI‐N87 cells at Atom Bioscience, sensitivity to 5F 203 was evident (IC_50_ ~ 24 nmol/L).

**Table 1:** Activity of 5F 203 against human gastric (and MCF‐7 breast) cancer cell lines

Cell lines		IC_50_ (Mean ± SD μmol/L)
MCF‐7		0.089 ± 0.027
MKN‐45	0.041^a^	0.090 ± 0.0076
ACS	0.039^a^	
SGC‐7901		>50
BGC‐823		>50
KATO Ш	>10^a^	>50
NCI‐N87	0.024^a^	1.54 ± 0.41

Note

Cells were seeded at 4 × 10^3^. Following 72‐h exposure to 5F 203, MTT assays were performed (maximum concentration examined = 50 μmol/L).

a Data generated by Atom Bioscience and Pharmaceutical Co. Ltd. Cells were seeded at 3 × 10^3^. Following 72‐h 5F 203 treatment, Alamar Blue assays were adopted to determine cell growth and viability; maximum concentration tested = 10 μmol/L.

Thus, within a gastric cancer cell line panel, 5F 203 demonstrates both selectivity (>250‐fold) and potent activity against MKN‐45 and ACS cells.

### 3.2 Intracellular distribution of AhR in gastric cancer cell lines

**Figure 2:**
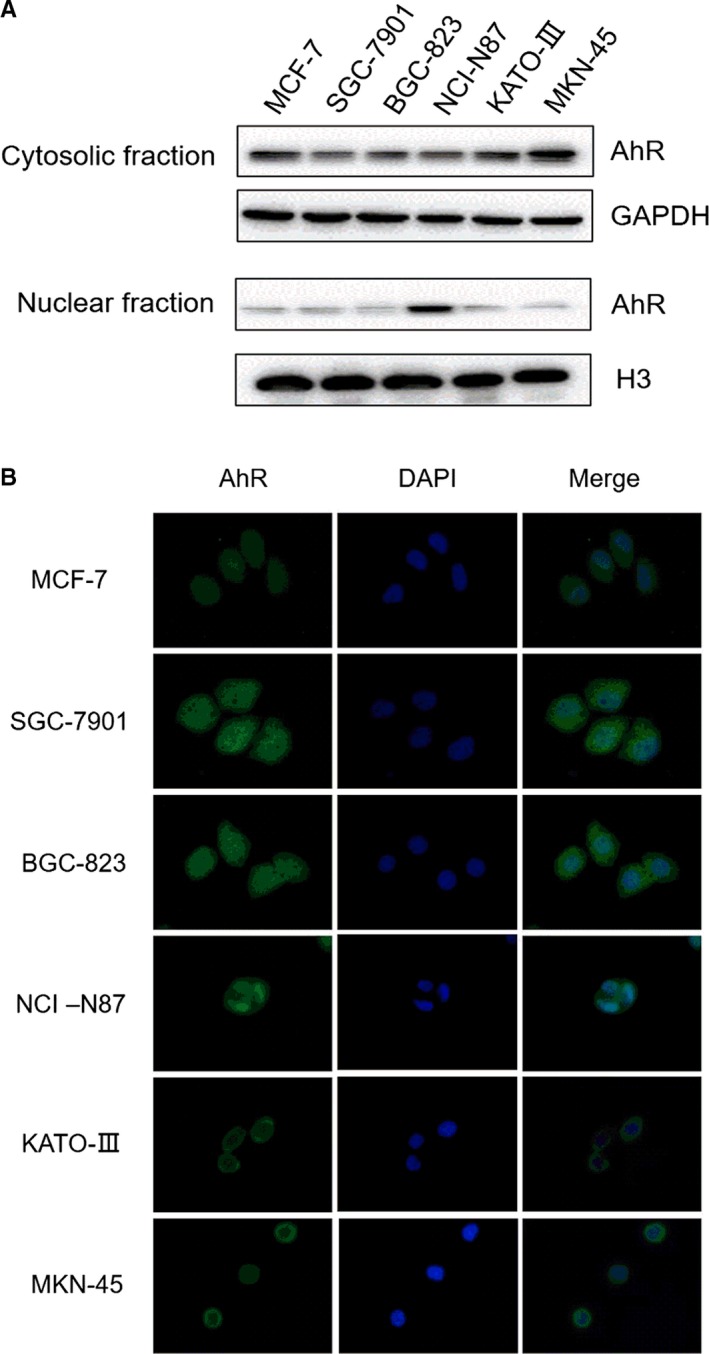
Intracellular distribution of AhR in gastric cancer cell lines. A, Gastric cancer cell lines were cultured, collected and extracted with Nuclear and Cytoplasmic Protein Extract ion Kit and subjected into immunoblot analyses. B, Immunofluorescence assay was performed to examine AhR localization

To investigate intracellular distribution of AhR, cell lysates were prepared following extraction using cytosolic and nuclear fraction kits, and subjected to Western blot. A substantial fraction of AhR was detected in the cytoplasmic fractions of KATO III and MKN‐45 lysates (Figure 1). In contrast, low levels of AhR were present in the cytoplasm of NCI‐N87 and BGC‐823 cells, and very faint AhR expression was detected in SGC‐7901 cells. Furthermore, the AhR localization in the cytoplasm was confirmed by immunofluorescence (Figure 2B). Interestingly, high levels of AhR were present in nuclei of NCI‐87 cells.

### 3.3 Induction of CYP mRNA by 5F 203 in MKN‐45 gastric cancer cell line

**Figure 3:**
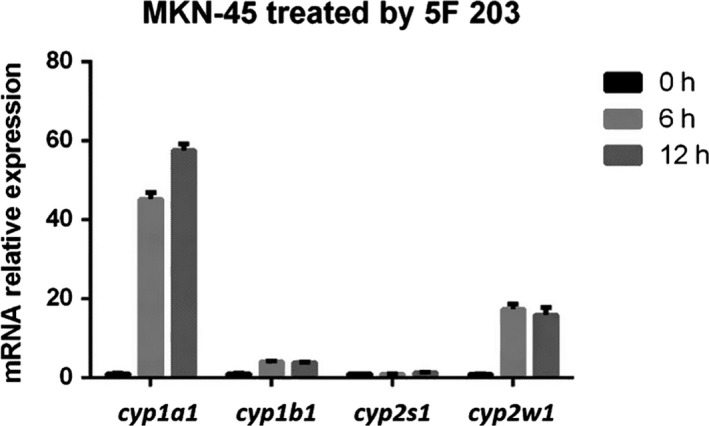
Induction of CYP1A1*,* CYP1B1, CYP2S1 and CYP2W1 gene expressions in cells treated with 1 μmol/L 5F 203. MKN‐45 cells were treated with 5F 203 (1 μmol/L) for 6 and 12 h, RNA was isolated, and real‐time PCR was performed to measure CYP1A1, CYP1B1, CYP2S1 and CYP2W1 mRNA levels

To investigate whether 5F 203 can induce CYP1A1, CYP1B1, CYP2S1 and CYP2W1 mRNA expression in sensitive MKN‐45 cells, MKN‐45 cells were treated with 5F 203 (1 μmol/L) for 6 and 12 hours and mRNA levels for these genes were measured by RT‐PCR. 5F 203 induced an increase in mRNA levels of both CYP1A1 and CYP2W1 in MKN‐45 cells (Figure 3). Treatment caused remarkable >50‐fold and ~20‐fold induction in CYP1A1 and CYP2W1 mRNA levels, respectively, compared with the control. Modest (5‐fold) induction of CYP1B1 mRNA was observed. However, the levels of CYP2S1 after treatment remained similar to control.

### 3.4 5F 203 induced AhR translocation and CYP1A1, CYP2W1 expression in MKN‐45 gastric cancer cell line

**Figure 4:**
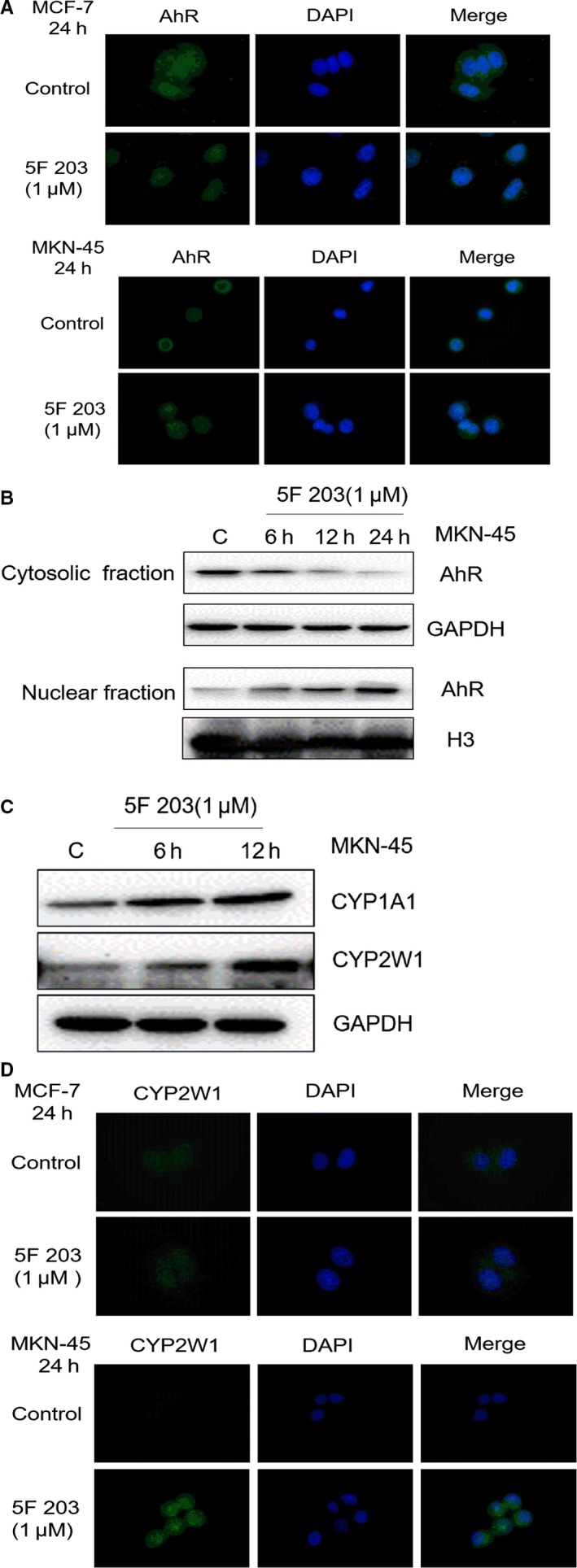
AhR translocation and induction of CYP1A1 and CYP2W1 in cells treated with 5F 203. A, MCF‐7 and MKN‐45 cells were treated with 5F 203 (1 μmol/L) for 24 h, and treated cells were fixed and double stained for AhR and DAPI as described under materials and methods. Stained cells were visualized on Nikon epifluorescence microscope. B, Treated cells were collected and lysed (Nuclear and Cytoplasmic Protein Extraction Kit; Beyotime P0028) in accordance with the manufacturer's instructions and subjected to immunoblot analysis. C, MNK‐45 cells were treated with 1 μmol/L 5F 203 for 6 and 12 h, and treated cells were collected, lysed and subjected to immunoblot analysis. D, MCF‐7 and MKN‐45 cells were treated with 5F 203 (1 μmol/L) for 24 h, treated cells were fixed and double stained for CYP2W1 and DAPI as described above, and immunofluorescence was visualized on Nikon epifluorescence microscope

To assess whether 5F 203 could activate the AhR signal transduction pathway, AhR translocation from cytoplasm to nucleus was monitored in MKN‐45 cells by immunofluorescence microscopy. In control cells (vehicle‐treated MCF‐7 and MKN‐45 cells), AhR is localized exclusively in the cytoplasm. However, after treatment with 5F 203 (1 μmol/L) for 24 hours, AhR had translocated completely to nucleus (Figure 4A). To confirm immunofluorescence studies, the effect of 5F 203 on the subcellular distribution of AhR protein was investigated by Western blot in sensitive MKN‐45 cells. After treatment with 5F 203 (1 μmol/L) for 6, 12 and 24 hours, AhR protein had translocated from cytoplasm to nuclei (Figure 4B).

Faint constitutive expression of CYP1A1 and CYP2W1 was detected in cytoplasm and nuclei of MNK‐45 gastric cancer cells. Following treatment of cells with 5F 203 (1 μmol/L) for 6 and 12 hours, enhanced CYP1A1 and CYP2W1 protein levels were expressed; induction of CYP1A1 and CYP2W1 could initially be detected after 6‐hour treatment of cells. Induction of CYP2W1 expression (by 1 μmol/L 5F 203; 24‐hour exposure) was further confirmed by immunofluorescence in MCF‐7 and MNK‐45 cells (Figure 4C,D).

### 3.5 5F 203 caused G2/M arrest, DNA damage and apoptosis in MKN‐45 gastric cancer cells

**Figure 5:**
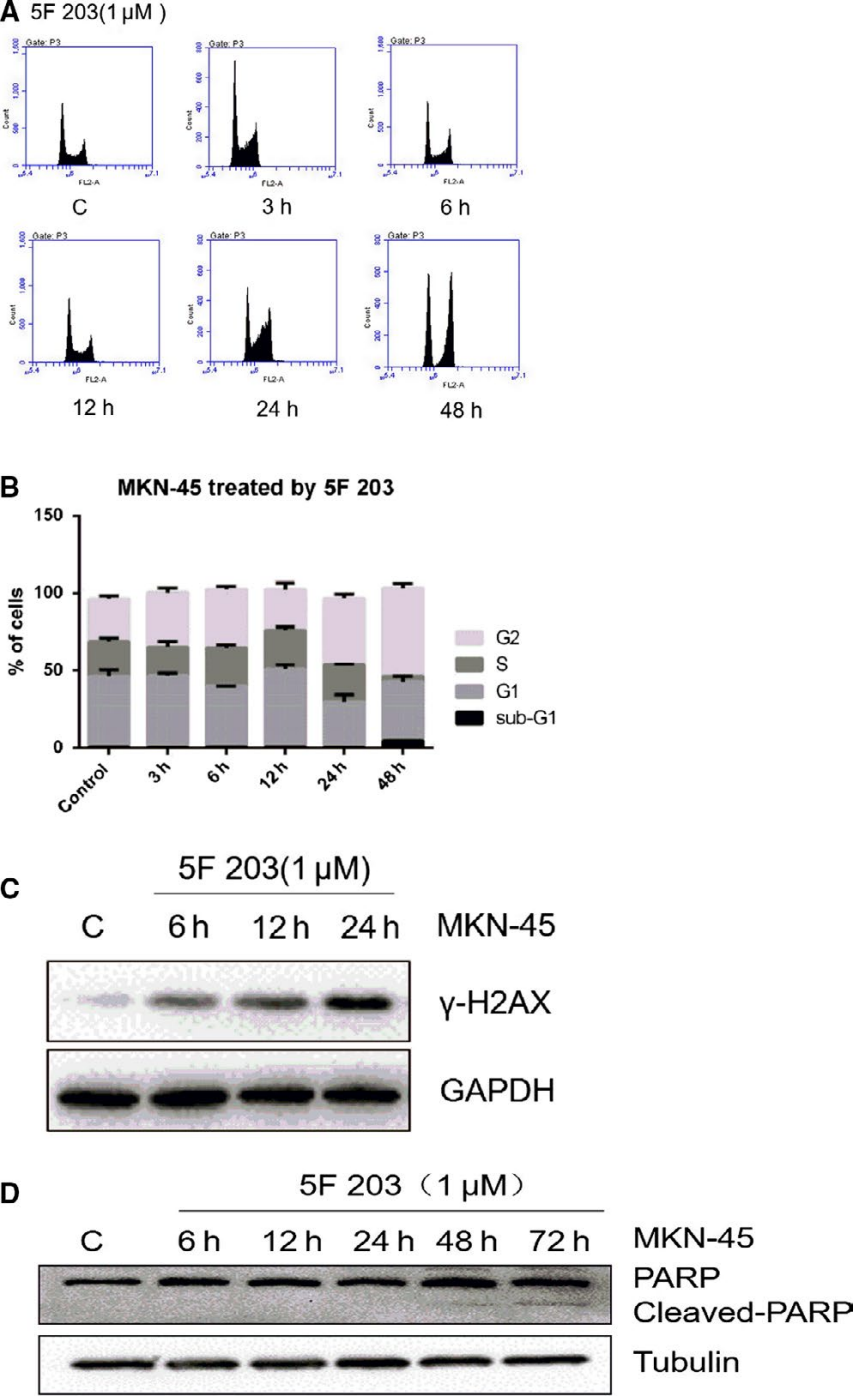
MNK‐45 cell cycle distribution, DNA damage induction and apoptosis after 5F 203 treatment. A, Representative DNA histograms of MNK‐45 cells, MNK‐45 cells were treated with 1 μmol/L 5F 203 for 3, 6, 12 and 24 h, and treated cells were collected and stained with propidium iodide and analysed by flow cytometry. B, Quantification of cell cycle distribution after 5F 203 treatment, experiments were performed in triplicate; 3 independent trials. C, Induction of DNA damage and apoptosis by 5F 203, detection of phosphorylated (γ) H2AX) and cleaved PARP by immunoblot analysis. MNK‐45 cells were treated with 1 μmol/L 5F 203 for 6, 12, 24 and 72 h. Treated cells were collected and subjected to immunoblot analysis

To investigate perturbations in cell cycle distribution after treatment of MKN‐45 cells with 5F 203, cells were treated with 5F 203 (1 μmol/L) for 3, 6, 12, 24 and 48 hours and subsequently processed for cell cycle analyses. As illustrated (Figure 5A,B), 5F 203 caused significant S‐G2/M arrest from 43.81% (control) to 60.9% at 24 hours. Significant accumulation of G2/M was detected from 15.26% (control) to 41.49% at 48 hours. In addition, H2AX phosphorylation (occurring a sites of DNA double‐strand breaks) was observed after 5F 203 (1 μmol/L; ≥6 hours; Figure 5C) treatment in the sensitive cell lines, indicating the presence of DNA damage in these cells. Immunoblot analysis showed that PARP cleavage occurred ≥48 hours after 5F 203 (1 μmol/L) treatment (Figure 5D).

### 3.6 5F 203 suppressed gastric tumour growth in vivo

**Figure 6:**
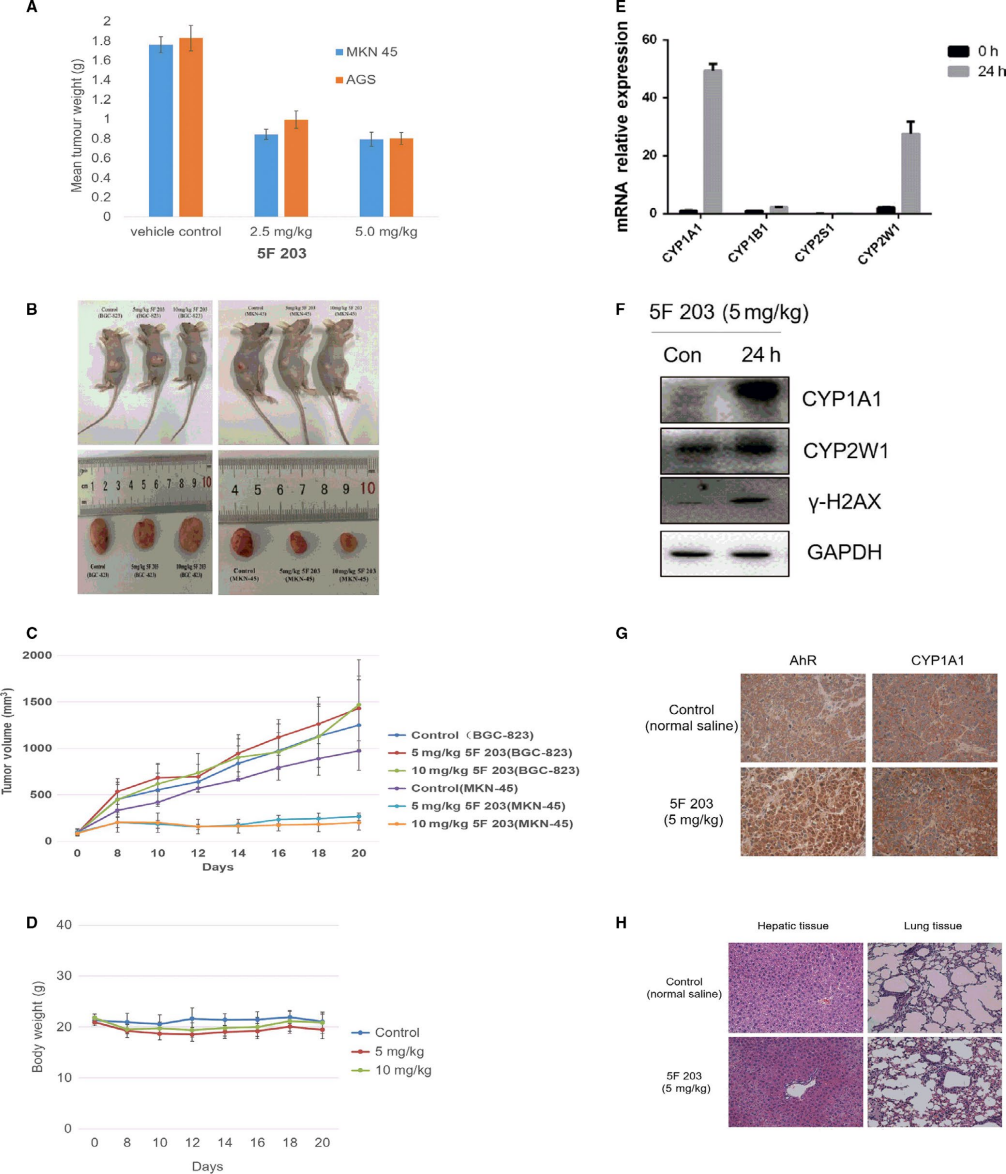
5F 203 suppressed MKN‐45 and AGS tumour growth in vivo. A, Reduction in mean MKN‐45 and AGS tumour weights following daily (days 0‐4; iv) treatment of mice with 5F 203^#^. B and C, MKN‐45 and BGC‐823 xenografts were transplanted in opposite flanks of the same mouse, and mice were treated with 5F 203 (5 mg/kg, 10 mg/kg; ip). B, Representative tumours are shown C, Effect of 5F 203 on the growth of MKN‐45 and BGC823 tumours. D, Bodyweight of mice after 5F 203 treatment. E, Selective CYP1A1 and CYP2W1 induction. MKN‐45 tumour‐bearing mice were treated with vehicle or 5 mg/kg 5F 203 ip, and tissues retrieved 24 h later. mRNA expression of CYP1A1, CYP1B1, CYP2S1 and CYP2W1 in tumours. F, Protein expression of CYP1A1, CYP2W1 and γ‐H2AX in tumours. G, Immunohistochemical analyses of AhR and CYP1A1 expression in tumours. H, H&E staining of lung and liver tissues

Independently conducted efficacy studies confirmed that 5F 203 inhibits gastric tumour growth in vivo. MKN‐45 and AGS xenograft growth was significantly inhibited by 5F 203 (2.5 mg/kg or 5 mg/kg administered iv on 5 consecutive days; *P* < .01; see SI). 5F 203 (5 mg/kg) inhibited tumour growth >50% as assessed by measurement of tumour volumes throughout the experiment and tumour weight at termination of the experiment (Figure 6A and Figure S1). To investigate the selective nature of gastric tumour growth inhibition caused by 5F 203 in vivo, MKN‐45 and BGC‐823 xenografts were transplanted in opposite flanks of the same mouse. As shown in Figure 6B,C, the growth of MKN‐45 growth was significantly suppressed after 5F 203 administration ip (5 mg/kg, 10 mg/kg), and the relative tumour volume in 5F 203 (5 mg/kg, 10 mg/kg) groups reduced by 72.55% and 79.30%, respectively, compared with the control group (*P* < .001). However, 5F 203 (5 mg/kg, 10 mg/kg) did not inhibit BGC‐823 tumour growth. The bodyweights of mice remained unchanged (Figure 6D), and no other observable side‐effects were detected (behaviour, appetite). Significant induction of CYP1A1 and CYP2W1 protein and mRNA expressions was demonstrated (Figure 6E,F) in homogenates of MKN‐45 tumours 24 hours after treatment of mice with 5F 203 (5 mg/kg, ip). The expression of CYP1B1 and CYP2S1 mRNA was not observed. In addition, γ‐H2AX protein expression was induced in MKN‐45 tumours 24 hours after mice were treated with 5F 203. Neither CYP1A1 nor CYP2W1 protein was detected in homogenates of MKN‐45 tumours recovered from vehicle‐treated mice. In BGC‐823 xenografts, CYP1A1 and CYP2W1 protein and mRNA expression were neither constitutive nor induced by treatment of mice with 5 mg/kg 5F 203.

Expression of AhR and CYP1A1 protein in MKN‐45 tumour tissue was analysed by immunohistochemistry. We found that AhR expression in vehicle‐treated mice was mainly in the cytoplasm, and AhR translocated into the nucleus after treatment with 5F 203. Furthermore, CYP1A1 protein was induced in the tumour tissues of mice after 5F 203 treatment (Figure 6G). Lung and liver tissues were recovered from animals 24 hours after treatment with 5F 203, and H&E staining revealed minor swelling of cells in the liver (Figure 6H).

## 4 DISCUSSION

In this communication, the potent and selective antitumour activity of 5F 203 (already described in breast, ovarian and renal cancer models) against gastric cancer cell lines MKN‐45 and AGS (IC_50_ value <100 nmol/L) has been revealed. In contrast, IC_50_ values >50 μmol/L were obtained in SGC‐7901, BCG‐823 and KATO III cell lines, revealing stark (>500‐fold) selectivity (Table 1). Treatment of MKN‐45 cells with 5F 203 triggered cell cycle arrest (Figure 5), H2AX phosphorylation indicative of DNA double‐strand breaks leading to initiation of apoptosis (Figure 5) strongly suggesting recognition of DNA‐damaging events and failure of repair leading to programmed cell death, selectively in MKN‐45 cells. Efficacy in vivo has also been demonstrated: 5F 203 significantly inhibited the growth of MKN‐45 (ip and iv administration routes) and AGS gastric xenografts. Moreover, selective efficacy was observed in mice‐bearing 5F 203‐sensitive (MKN‐45) and 5F 203‐insensitive (BGC‐823) xenografts transplanted s.c. in opposite flanks.

The mechanism of action of 5F 203 is distinct from anticancer agents in the clinical arena. A potent AhR ligand,^20^ this lipophilic agent readily diffuses across cell membranes and binds with high affinity to cytosolic AhR. Translocation to the nucleus and binding to xenobiotic response elements induced transcription of genes within the AhR battery.^21^ In breast, ovarian and renal cancer panels, sensitivity to aminophenylbenzothiazols including 5F 203 correlated with induction of CYP1A1 gene transcription^9^ and CYP1A1 protein expression.^3^

Subsequently, in colorectal carcinoma cell lines, expressing neither constitutive nor inducible CYP1A1,^14^ CYP2W1 was found to bioactivate 5F 203 and structurally related 2‐(3,4‐dimethoxyphenyl)‐5‐fluorobenzothiazole (GW 610); stable knockdown of CYP2W1 gene led to significant loss of benzothiazole (GW 610, 5F 203) activity in KM12 and HCC 2998 CRC cell lines.^22^

It was thereafter irrefutably demonstrated that CYP1A1 and CYP2W1 are able to catalyse production of 5F 203‐derived hydroxylamine, and guanine DNA adducts presumably *via* nitrenium species production, underpinning the selective antitumour cytotoxic activity of 5F 203.^6^ 5F 203 treatment consequently resulted in lethal DNA adducts, double‐strand breaks and selective cancer cell death.^8^

Expression of CYP2S1 mRNA, whose protein product is able to catalyse conversion of the 5F 203‐derived hydroxylamine back to its parent amine^6^ and may diminish its antitumour activity, was negligibly affected following exposure of MKN‐45 cells to 5F 203.

Thus, PD biomarkers of sensitivity to/activity of 5F 203 activity in in vitro, in vivo and ex vivo breast ovarian, renal and colon cancer models include cytosolic AhR expression and translocation,^11^ CYP 1A1 induction,^3^, ^10^, ^23^ CYP2W1 expression,^14^, ^22^ DNA adducts' generation^7^, ^14^ and DNA strand breaks.^7^, ^8^

In vitro, strong cytosolic AhR expression together with powerful (>50‐fold) induction of CYP1A1 mRNA and CYP1A1 protein by 5F 203 was detected in MKN‐45 lysates, as well as ~20‐fold induction of CYP2W1 mRNA and subsequent protein expression. In contrast, in all other gastric cancer cell lines within this panel, CYP1A1 induction was not evident (results not shown). In BGC‐823, KATO III, NCI‐N87 and SGC‐7901 cytosolic and nuclear lysate fractions, AhR expression appeared low. We may speculate that in cells expressing no/low cytosolic AhR, there is limited capacity for cytosolic 5F 203‐AhR binding, minimal translocation to nuclei, and therefore, AhR signal transduction is not triggered by 5F 203. Intriguingly however, NCI‐87 cells expressed high constitutive AhR in nuclear fractions. Further investigations are necessary to understand molecular mechanisms underpinning 5F 203 insensitivity in this cell line. It is likely that cytosolic AhR and pathway activation are fundamental for 5F 203 activity. Notably, NCI‐N87 expressed low cytosolic AhR levels and was the only Kunming cell line other than MKN‐45 to express cytosolic and nuclear CYP2W1. 5F 203 very weakly inhibited Kunming NCI‐N87 cell growth with an IC_50_ value of 22.63 μmol/L; intriguingly, however, Atom Bioscience NCI‐N87 cells demonstrated exquisite sensitivity to 5F 203 (GI_50_ 24.3 nmol/L^#^). A similar paradox has been observed previously in HCC2998 CRC cells yielding dichotomous GI_50_ values <10 nmol/L or >10 μmol/L (with no intermediate values). Neither constitutive nor inducible CYP1A1 was detected in HCC2998 cells, and however, CYP2W1 protein was expressed. It would be interesting to determine whether NCI‐N87 cells are responsive to GW 610, as CYP 2W1‐catalysed oxidation of GW 610 is 5‐fold more efficient that the CYP 1A1‐catalysed bioactivation.^6^ Structurally related GW 610^6^, ^14^, ^24^ demonstrated potent activity against mammary, ovarian carcinoma (demonstrating inducible CYP1A1) and also CRC cell lines expressing CYP2W1.

In sensitive breast and ovarian cell lines, following activation of AhR signalling and induction of CYP1A1, DNA adducts are generated; similarly, CYP (1A1/2W1)‐catalysed activation of GW610 leads to generation of DNA adducts in sensitive cell phenotypes. DNA adducts precipitate lethal DNA DSBs at which sites histone H2AX is phosphorylated. γH2AX appeared in a time‐dependent manner (≥6 hours) after treatment of MKN‐45 cells with 1 μmol/L 5F 203; cell cycle perturbations appear to corroborate accumulation of DNA damage as events accumulate significantly in S‐ (24 hours) and G2/M‐cell cycle phases, presumably as cells attempt to repair DNA. Emergence of pre‐G1 events indicative of apoptotic populations implies repair failure. PARP cleavage ≥48 hours confirms the apoptotic fate of MKN‐45 cells exposed to 5F 203.

Analyses of MKN‐45 tumours excised from mice exposed to 5F 203 (or vehicle) exposed in vivo PD biomarkers of sensitivity/activity (AhR expression, induction of CYP1A1, CYP2W1 mRNA and protein expression, γH2AX) in gastric cancer corroborating a role for AhR signal transduction in the mechanism of action of 5F 203.

Thus, for sensitivity to antitumour benzothiazoles to manifest, the combination of cytosolic AhR and inducible CYP1A1 or CYP2W1 expression seems to be required.

Initial developmental expression and re‐expression in certain tumours of CYP 2W1 implicate roles in growth and tumorigenesis. Indeed, CYP2W1 is known to be a poor prognostic marker in CRC and expression in metastatic disease is enhanced compared with primary malignant sites.^25^, ^26^ Tumour‐specific expression of CYP2W1 infers potential as a putative molecular drug target, able to bioactivate anticancer prodrug candidates at the tumour site (minimizing systemic exposure to active species). Antitumour benzothiazoles, for example 5F 203, are bioactivated by CYP2W1 and CYP1A1^6^—producing dGuo adducts responsible for cytotoxicity. Discovery of CYP 2W1 expression in gastric and hepatocellular carcinomas extends the spectrum of cancer phenotypes potentially demonstrating sensitivity to potent, selective antitumour benzothiazoles.

The demonstration herein of 5F 203 activity against gastric cancer models expressing cytosolic AhR and inducible CYP1A1 and CYP2W1 suggests potential for the development of antitumour benzothiazoles for treatment of certain gastric cancers which pose a huge morbidity burden, represent an unmet clinical need, and for which there is currently no standard of care.

## CONFLICT OF INTEREST

Atom Bioscience, JZ, MFGS and TDB are named on a patent application submitted in 2018 describing the use of 5F 203 or a pharmaceutically acceptable salt thereof in the manufacture of a medicament for treating or preventing gastric cancer. The authors confirm that there are no conflicts of interest.

## AUTHORS' CONTRIBUTIONS

Wang YL, Liu Y, Cheng X, Yang Y performed the experiments and statistical analysis. Stevens M designed and provided material support of 2‐(4‐amino‐3‐methylphenyl)‐5‐fluorobenzothiazole (5F 203). J Zhang and TD Bradshaw designed the experiments and wrote the paper. Shi DF provided the materials. Luo Y and Tang T provided the research material and equipment. Bradshaw T and Zhang JH revised the manuscript. Bradshaw T and Zhang JH gave the final approval of the version to be published. All authors read and approved the version of final manuscript.

## Data Availability

Data generated by research reported herein will be made openly and publicly available upon publication. This is part 36 in the series ‘Antitumour Benzothiazoles’.
