# Supplementary material for: The antitumour activity of 2‐(4‐amino‐3‐methylphenyl)‐5‐fluorobenzothiazole in human gastric cancer models is mediated by AhR signalling
Source: J Cell Mol Med. 2019 Dec 25;24(2):1750–9. doi: 10.1111/jcmm.14869 (PMC6991636; doi:10.1111/jcmm.14869)
Supplement: Supplementary file 1 [file JCMM-24-1750-s001.docx]

Supplementary Information.

**The antitumor activity of 2-(4-Amino-3-methylphenyl)-5-fluorobenzothiazole in human gastric cancer models is mediated by AhR receptor signaling**

Yuling Wang^1#^, Yin Liu^1#^ _,_ Tao Tang^1*^_,_ Ying Luo^1^, Malcolm F.G. Stevens^2^, Xi Cheng^3^, Yan Yang^3^, Dongfang Shi^3^, Jihong Zhang^1*^, Tracey D. Bradshaw^2*^

Materials and Methods for examination of antitumour efficacy of 5F 203 against MKN-45 and AGS gastric cancer models.

Materials

5F 203 was synthesised by Jiangsu Atom Bioscience and Pharmaceutical Company, Ltd.

PEG400 was purchased from Chengdu Kelong Factory (lot no. 20180412); NaCl (physiological saline) was purchased from Hebei Tiancheng Pharmaceutiical Company, Ltd (lot no. A17111105); DMSO was obtained from MP Biomedicals (lot no. Q6949).

Cell lines were obtained from the Cell Resource Centre of Shanghai Institutes for Biological Sciences of the Chinese Academy of Sciences and culture medium (AGS, RPMI 1640; MKN-45, DMEM) supplemented with 10% FBS.

Athymic nude mice (BALB/c-nu, female, SPF), 6-8 weeks old and weighing 18-20 g were purchased from Changzhou Cavens Inc. Experimental animal production license SYXK (SU) 2017-0007; lot no. 20182073.

Methods

For the low dose group: 2.5 mg/kg delivered i.v.; 2.0 mg 5F 203 was dissolved in injection solvent (PEG400 25%, DMSO 2.5%, saline 72.5%). The final concentration of i.v. solution was 0.25 mg/mL; the volume injected was 0.2 mL/20 g body weight. For animals receiving 5 mg/kg, 4.0 mg 5F 203 was dissolved in i.v. injection solvent (PEG400, 50%, DMSO 5%, saline 45%). The final concentration of i.v. solution was 0.5 mg/mL; the volume injected was 0.2 mL/20 g body weight.

AGS and MKN-45 tumours, in log growth phase were isolated from nude mice and cell suspensions prepared. Cells (5 x 10^6^) were inoculated s.c. into 60 nude mice at the site of the right axilla. Lengths and widths of growing xenografted tumours were measures using a Vernier caliper. Mice (56) possessing tumours with good uniformity (100 mm^3^) were randomly divided into 7 groups containing 8 mice per group. Test agent groups received 5F 203 by i.v. injection on 5 consecutive days (designated days 0-4 inclusive). Mice were observed for 20 days. Tumour growth / size was monitored every 2 days by measuring the length (a) and width (b) with calipers and calculating the tumour volume (TV) using the formula:

TV = 0.5 x a x b^2^

Upon sacrifice, tumours were isolated surgically and weighed.

Data are expressed as means ± SD. Multiple groups were compared with one-way ANOVA; 2 groups were compared using Dunnett`s test using GraphPad Prism 5. Statistical significance was concluded when *p*<0.05; ***p*<0.01.

Results

Figure S1. The Figure show the antitumour efficacy of 5F 203 (2.5 and 5.0 mg/kg delivered i.v.) against A) MNK-45 and B) AGS xenograft growth. Tumour sizes were measured by calipers and volumes calculated. Means ± SD n=8; *p*<0.01.

A


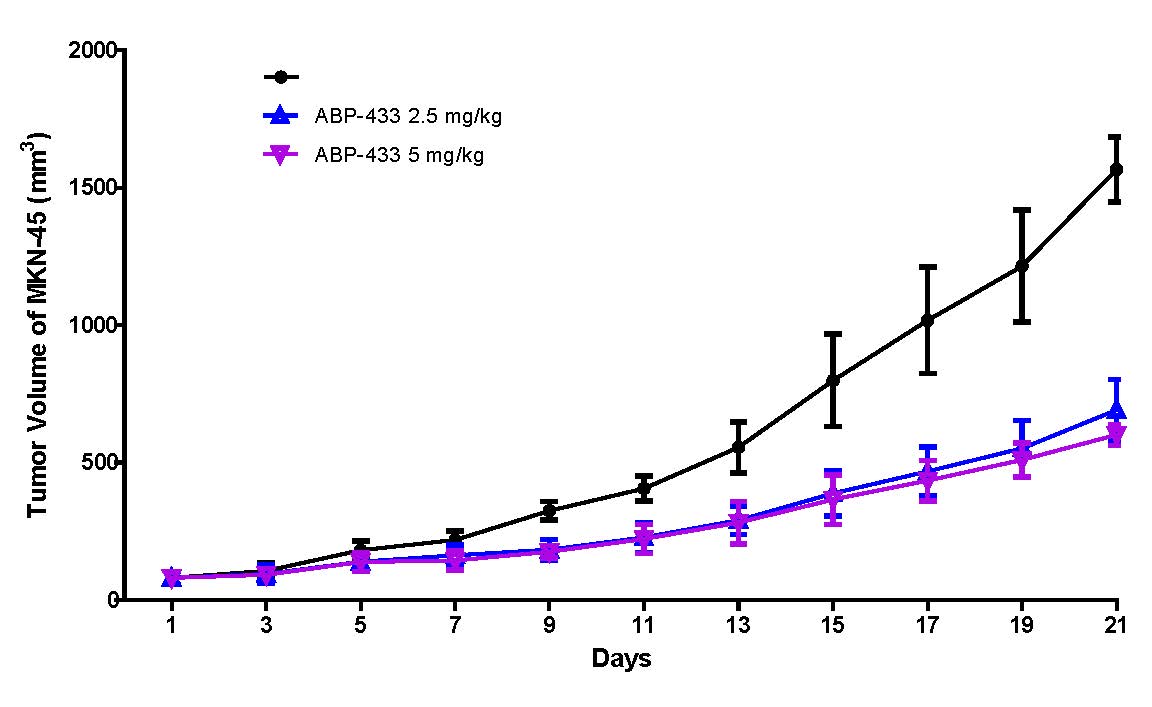


**Control**

**5F 203 2.5 mg/kg**

**5F 203 5 mg/kg**

B

B


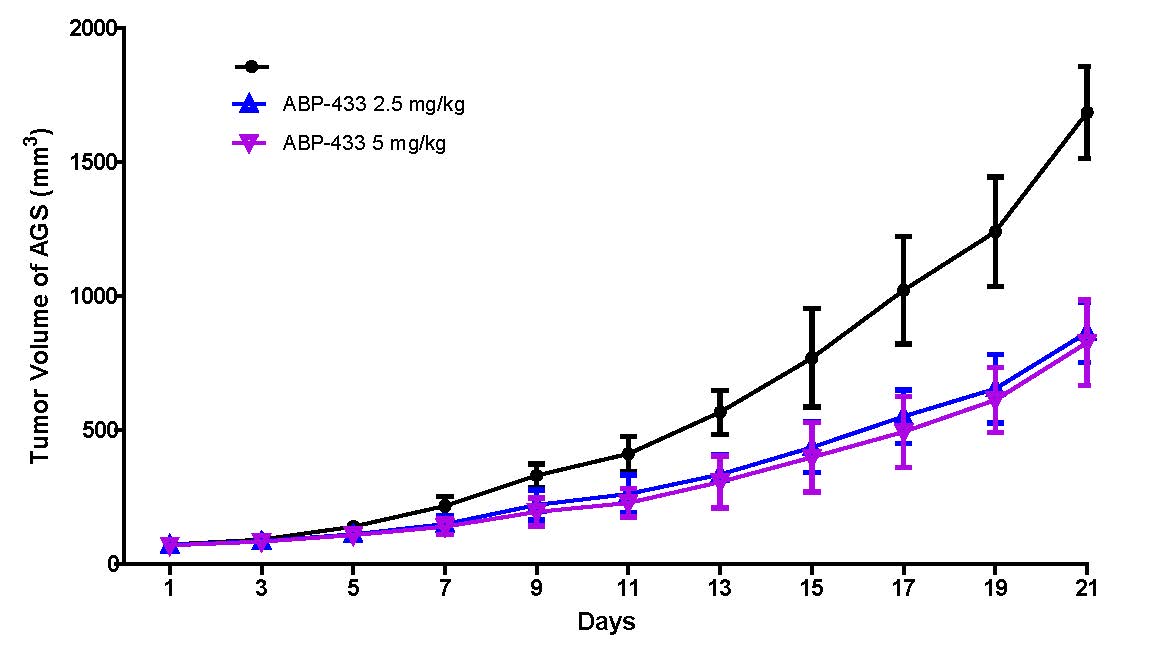


**Control**

**5F 203 2.5 mg/kg**

**5F 203 5 mg/kg**
