# Supplementary material for: The antitumour activity of 2‐(4‐amino‐3‐methylphenyl)‐5‐fluorobenzothiazole in human gastric cancer models is mediated by AhR signalling
Source: J Cell Mol Med. 2019 Dec 25;24(2):1750–9. doi: 10.1111/jcmm.14869 (PMC6991636; doi:10.1111/jcmm.14869)
Supplement: Supplementary file 2 [file JCMM-24-1750-s002.pdf]

National Cancer Institute Developmental Therapeutics Program Dose Response Curves	NSC: 703786 / 4	SSPL: Q48P	EXP. ID: 0301NS02
	Report Date: September 27, 2007	Test Date: January 27, 2003	

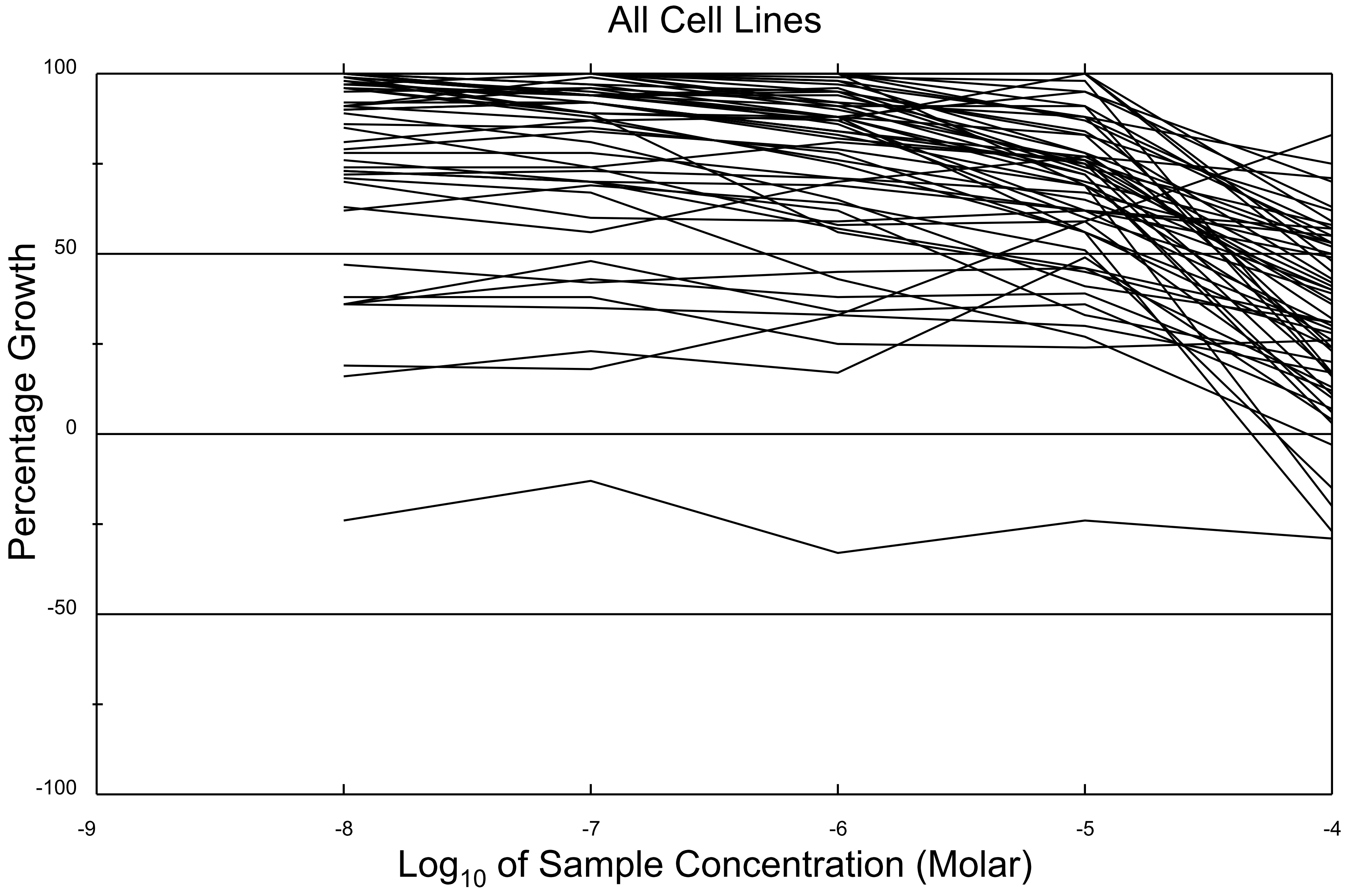

National Cancer Institute Developmental Therapeutics Program Dose Response Curves	NSC: 703786 / 4	SSPL: Q48P	EXP. ID: 0301NS02
	Report Date: September 27, 2007	Test Date: January 27, 2003	

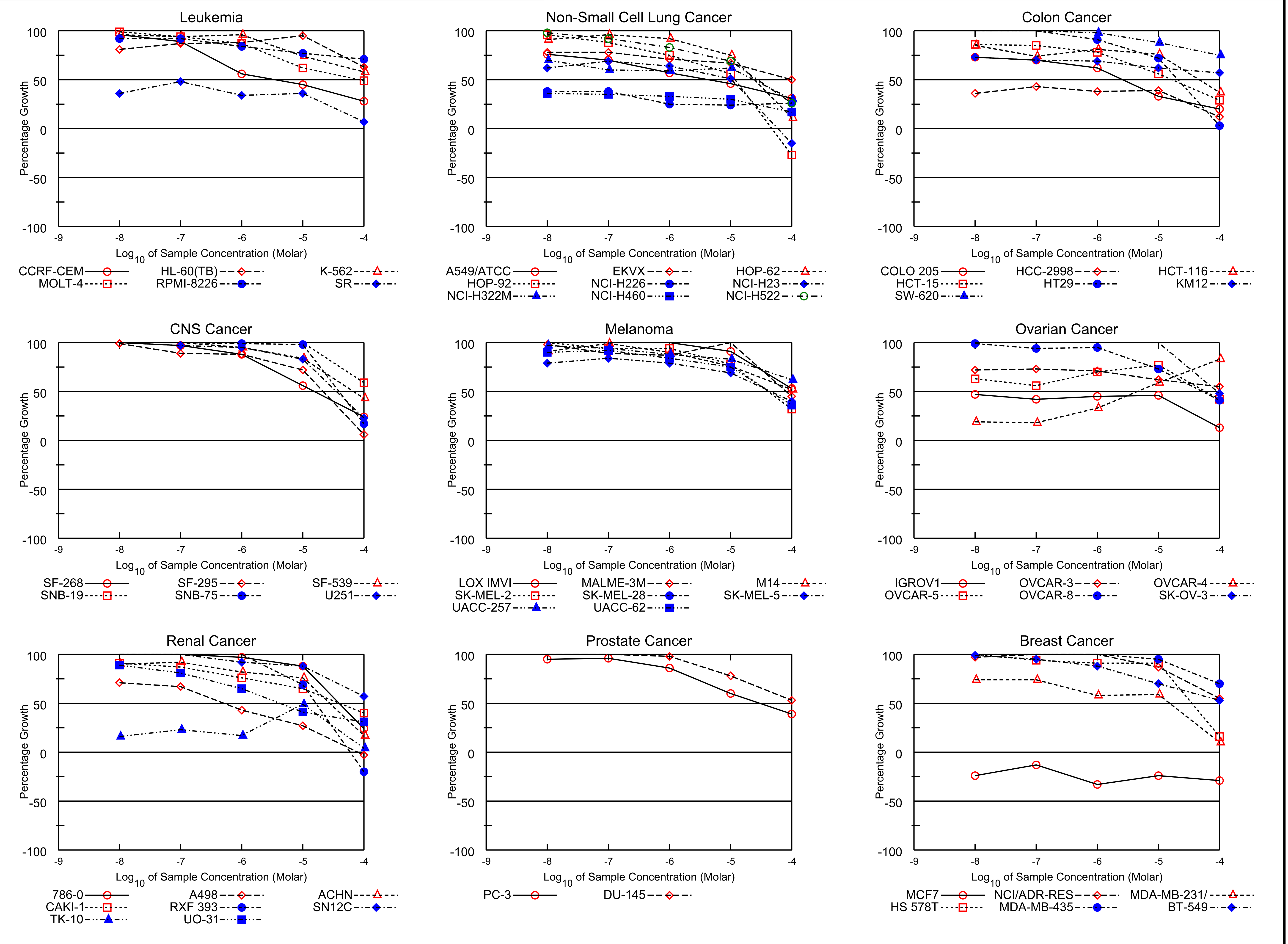

National Cancer Institute Developmental Therapeutics Program
In-Vitro Testing Results

NSC : 703786 / 4	Experiment ID : 0301NS02	Test Type : 08	Units : Molar
Report Date : September 27, 2007	Test Date : January 27, 2003	QNS :	MC :
COMI : KS58-143-3	Stain Reagent : SRB Dual-Pass Related	SSPL : Q48P	

Log10 Concentration

	Time		Mean Optical Densities					Percent Growth							
Panel/Cell Line	Zero	Ctrl	-8.0	-7.0	-6.0	-5.0	-4.0	-8.0	-7.0	-6.0	-5.0	-4.0	GI50	TGI	LC50
Leukemia															
CCRF-CEM	0.344	1.786	1.725	1.629	1.148	0.996	0.754	96	89	56	45	28	3.52E-6	> 1.00E-4	> 1.00E-4
HL-60(TB)	0.183	0.643	0.557	0.583	0.587	0.622	0.472	81	87	88	95	63	> 1.00E-4	> 1.00E-4	> 1.00E-4
K-562	0.325	2.626	2.528	2.478	2.537	2.018	1.663	96	94	96	74	58	> 1.00E-4	> 1.00E-4	> 1.00E-4
MOLT-4	0.219	1.175	1.168	1.115	1.049	0.816	0.690	99	94	87	62	49	8.74E-5	> 1.00E-4	> 1.00E-4
RPMI-8226	0.518	1.415	1.343	1.347	1.274	1.211	1.156	92	92	84	77	71	> 1.00E-4	> 1.00E-4	> 1.00E-4
SR	0.206	0.596	0.347	0.393	0.338	0.347	0.232	36	48	34	36	7	< 1.00E-8	> 1.00E-4	> 1.00E-4
Non-Small Cell Lung Cancer															
A549/ATCC	0.155	0.715	0.583	0.548	0.473	0.414	0.329	76	70	57	46	31	4.37E-6	> 1.00E-4	> 1.00E-4
EKVX	0.397	1.045	0.905	0.901	0.856	0.830	0.724	78	78	71	67	50	> 1.00E-4	> 1.00E-4	> 1.00E-4
HOP-62	0.335	1.068	1.003	1.040	1.013	0.884	0.414	91	96	92	75	11	2.44E-5	> 1.00E-4	> 1.00E-4
HOP-92	0.702	1.075	1.061	1.030	0.983	0.911	0.514	96	88	75	56	-27	1.18E-5	4.75E-5	> 1.00E-4
NCI-H226	0.953	1.877	1.306	1.308	1.181	1.175	1.193	38	38	25	24	26	< 1.00E-8	> 1.00E-4	> 1.00E-4
NCI-H23	0.372	1.112	0.829	0.880	0.847	0.748	0.318	62	69	64	51	-15	1.03E-5	5.97E-5	> 1.00E-4
NCI-H322M	0.504	0.958	0.822	0.776	0.773	0.785	0.640	70	60	59	62	30	2.34E-5	> 1.00E-4	> 1.00E-4
NCI-H460	0.322	1.728	0.830	0.810	0.784	0.748	0.567	36	35	33	30	17	< 1.00E-8	> 1.00E-4	> 1.00E-4
NCI-H522	0.608	1.508	1.490	1.438	1.359	1.233	0.843	98	92	83	69	26	2.81E-5	> 1.00E-4	> 1.00E-4
Colon Cancer															
COLO 205	0.626	1.465	1.238	1.210	1.144	0.901	0.794	73	70	62	33	20	2.54E-6	> 1.00E-4	> 1.00E-4
HCC-2998	0.414	0.902	0.592	0.625	0.602	0.604	0.475	36	43	38	39	12	< 1.00E-8	> 1.00E-4	> 1.00E-4
HCT-116	0.120	0.909	0.790	0.705	0.759	0.719	0.413	85	74	81	76	37	4.65E-5	> 1.00E-4	> 1.00E-4
HCT-15	0.265	1.401	1.247	1.232	1.147	0.903	0.597	86	85	78	56	29	1.69E-5	> 1.00E-4	> 1.00E-4
HT29	0.739	0.981	0.991	1.032	0.958	0.912	0.747	104	121	91	72	3	2.07E-5	> 1.00E-4	> 1.00E-4
KM12	0.306	1.538	1.208	1.167	1.153	1.064	1.005	73	70	69	62	57	> 1.00E-4	> 1.00E-4	> 1.00E-4
SW-620	0.137	0.973	1.004	0.987	0.958	0.875	0.761	104	102	98	88	75	> 1.00E-4	> 1.00E-4	> 1.00E-4
CNS Cancer															
SF-268	0.605	1.251	1.289	1.232	1.171	0.969	0.758	106	97	88	56	24	1.56E-5	> 1.00E-4	> 1.00E-4
SF-295	0.602	1.709	1.695	1.593	1.582	1.400	0.669	99	89	88	72	6	2.16E-5	> 1.00E-4	> 1.00E-4
SF-539	0.430	1.407	1.420	1.437	1.356	1.247	0.852	101	103	95	84	43	6.78E-5	> 1.00E-4	> 1.00E-4
SNB-19	0.652	1.090	1.173	1.138	1.100	1.151	0.910	119	111	102	114	59	> 1.00E-4	> 1.00E-4	> 1.00E-4
SNB-75	0.487	1.098	1.113	1.140	1.092	1.085	0.591	102	107	99	98	17	3.91E-5	> 1.00E-4	> 1.00E-4
U251	0.187	1.167	1.162	1.137	1.121	1.003	0.410	100	97	95	83	23	3.54E-5	> 1.00E-4	> 1.00E-4
Melanoma															
LOX IMVI	0.155	1.546	1.704	1.606	1.656	1.418	0.887	111	104	108	91	53	> 1.00E-4	> 1.00E-4	> 1.00E-4
MALME-3M	0.653	1.114	1.105	1.062	1.056	1.116	0.862	98	89	87	100	45	8.19E-5	> 1.00E-4	> 1.00E-4
M14	0.268	0.927	0.868	0.918	0.859	0.771	0.608	91	99	90	76	52	> 1.00E-4	> 1.00E-4	> 1.00E-4
SK-MEL-2	0.153	0.497	0.514	0.477	0.475	0.420	0.262	105	94	94	78	32	3.99E-5	> 1.00E-4	> 1.00E-4
SK-MEL-28	0.366	0.622	0.708	0.632	0.661	0.691	0.701	133	104	115	127	131	> 1.00E-4	> 1.00E-4	> 1.00E-4
SK-MEL-5	0.307	1.374	1.153	1.203	1.147	1.040	0.735	79	84	79	69	40	4.51E-5	> 1.00E-4	> 1.00E-4
UACC-257	0.263	1.753	1.702	1.667	1.580	1.495	1.185	97	94	88	83	62	> 1.00E-4	> 1.00E-4	> 1.00E-4
UACC-62	0.538	1.749	1.629	1.650	1.551	1.450	0.968	90	92	84	75	36	4.33E-5	> 1.00E-4	> 1.00E-4
Ovarian Cancer															
IGROV1	0.163	0.907	0.516	0.477	0.500	0.504	0.258	47	42	45	46	13	< 1.00E-8	> 1.00E-4	> 1.00E-4
OVCAR-3	0.428	1.316	1.066	1.075	1.060	0.976	0.913	72	73	71	62	55	> 1.00E-4	> 1.00E-4	> 1.00E-4
OVCAR-4	0.452	0.862	0.532	0.527	0.586	0.694	0.793	19	18	33	59	83	.	> 1.00E-4	> 1.00E-4
OVCAR-5	0.470	1.089	0.860	0.819	0.901	0.949	0.729	63	56	70	77	42	5.86E-5	> 1.00E-4	> 1.00E-4
OVCAR-8	0.310	1.064	1.057	1.020	1.028	0.857	0.617	99	94	95	73	41	5.09E-5	> 1.00E-4	> 1.00E-4
SK-OV-3	0.516	0.716	0.760	0.770	0.738	0.758	0.613	122	127	111	121	48	9.50E-5	> 1.00E-4	> 1.00E-4
Renal Cancer															
786-0	0.383	1.580	1.680	1.721	1.543	1.438	0.673	108	112	97	88	24	3.95E-5	> 1.00E-4	> 1.00E-4
A498	0.673	1.294	1.116	1.089	0.942	0.841	0.651	71	67	43	27	-3	5.21E-7	7.76E-5	> 1.00E-4
ACHN	0.278	1.289	1.185	1.213	1.109	1.044	0.455	90	92	82	76	17	2.76E-5	> 1.00E-4	> 1.00E-4
CAKI-1	0.581	1.619	1.526	1.489	1.371	1.253	0.993	91	87	76	65	40	3.87E-5	> 1.00E-4	> 1.00E-4
RXF 393	0.608	0.859	0.888	0.882	0.864	0.783	0.485	111	109	102	69	-20	1.65E-5	5.94E-5	> 1.00E-4
SN12C	0.521	1.267	1.299	1.349	1.207	1.179	0.946	104	111	92	88	57	> 1.00E-4	> 1.00E-4	> 1.00E-4
TK-10	0.655	1.398	0.775	0.830	0.784	1.017	0.687	16	23	17	49	4	< 1.00E-8	> 1.00E-4	> 1.00E-4
UO-31	0.414	1.774	1.618	1.516	1.292	0.973	0.836	89	81	65	41	31	4.16E-6	> 1.00E-4	> 1.00E-4
Prostate Cancer															
PC-3	0.609	2.514	2.421	2.435	2.249	1.755	1.347	95	96	86	60	39	2.97E-5	> 1.00E-4	> 1.00E-4
DU-145	0.359	1.356	1.397	1.377	1.332	1.136	0.887	104	102	98	78	53	> 1.00E-4	> 1.00E-4	> 1.00E-4
Breast Cancer															
MCF7	0.539	1.561	0.411	0.471	0.364	0.412	0.382	-24	-13	-33	-24	-29	< 1.00E-8	< 1.00E-8	> 1.00E-4
NCI/ADR-RES	0.379	1.026	1.006	1.031	1.054	0.945	0.736	97	101	104	87	55	> 1.00E-4	> 1.00E-4	> 1.00E-4
MDA-MB-231/ATCC	0.355	0.867	0.733	0.733	0.654	0.657	0.406	74	74	58	59	10	1.52E-5	> 1.00E-4	> 1.00E-4
HS 578T	0.636	1.517	1.541	1.461	1.440	1.440	0.776	103	94	91	91	16	3.52E-5	> 1.00E-4	> 1.00E-4
MDA-MB-435	0.540	2.050	2.112	2.127	2.092	1.968	1.592	104	105	103	95	70	> 1.00E-4	> 1.00E-4	> 1.00E-4
BT-549	0.439	0.964	0.958	0.940	0.902	0.808	0.717	99	95	88	70	53	> 1.00E-4	> 1.00E-4	> 1.00E-4

National Cancer Institute Developmental Therapeutics Program
Mean Graphs

NSC : 703786 / 4 | Units : Molar | SSPL : Q48P | EXP. ID : 0301NS02

Report Date : September 27, 2007 | Test Date : January 27, 2003

Panel/Cell Line	Log_{10}GI50	Log_{10}TGI	Log_{10}LC50
**Leukemia**			
CCRF-CEM	-5.45	> -4.00	> -4.00
HL-60(TB)	> -4.00	> -4.00	> -4.00
K-562	> -4.00	> -4.00	> -4.00
MOLT-4	-4.06	> -4.00	> -4.00
RPMI-8226	> -4.00	> -4.00	> -4.00
SR	< -8.00	> -4.00	> -4.00
**Non-Small Cell Lung Cancer**			
A549/ATCC	-5.36	> -4.00	> -4.00
EKVX	> -4.00	> -4.00	> -4.00
HOP-62	-4.61	> -4.00	> -4.00
HOP-92	-4.93	-4.32	> -4.00
NCI-H226	< -8.00	> -4.00	> -4.00
NCI-H23	-4.99	-4.22	> -4.00
NCI-H322M	-4.63	> -4.00	> -4.00
NCI-H460	< -8.00	> -4.00	> -4.00
NCI-H522	-4.55	> -4.00	> -4.00
**Colon Cancer**			
COLO 205	-5.60	> -4.00	> -4.00
HCC-2998	< -8.00	> -4.00	> -4.00
HCT-116	-4.33	> -4.00	> -4.00
HCT-15	-4.77	> -4.00	> -4.00
HT29	-4.69	> -4.00	> -4.00
KM12	> -4.00	> -4.00	> -4.00
SW-620	> -4.00	> -4.00	> -4.00
**CNS Cancer**			
SF-268	-4.81	> -4.00	> -4.00
SF-295	-4.67	> -4.00	> -4.00
SF-539	-4.17	> -4.00	> -4.00
SNB-19	> -4.00	> -4.00	> -4.00
SNB-75	-4.41	> -4.00	> -4.00
U251	-4.45	> -4.00	> -4.00
**Melanoma**			
LOX IMVI	> -4.00	> -4.00	> -4.00
MALME-3M	-4.09	> -4.00	> -4.00
M14	> -4.00	> -4.00	> -4.00
SK-MEL-2	-4.40	> -4.00	> -4.00
SK-MEL-28	> -4.00	> -4.00	> -4.00
SK-MEL-5	-4.35	> -4.00	> -4.00
UACC-257	> -4.00	> -4.00	> -4.00
UACC-62	-4.36	> -4.00	> -4.00
**Ovarian Cancer**			
IGROV1	< -8.00	> -4.00	> -4.00
OVCAR-3	> -4.00	> -4.00	> -4.00
OVCAR-4		> -4.00	> -4.00
OVCAR-5	-4.23	> -4.00	> -4.00
OVCAR-8	-4.29	> -4.00	> -4.00
SK-OV-3	-4.02	> -4.00	> -4.00
**Renal Cancer**			
786-0	-4.40	> -4.00	> -4.00
A498	-6.28	-4.11	> -4.00
ACHN	-4.56	> -4.00	> -4.00
CAKI-1	-4.41	> -4.00	> -4.00
RXF 393	-4.78	-4.23	> -4.00
SN12C	> -4.00	> -4.00	> -4.00
TK-10	< -8.00	> -4.00	> -4.00
UO-31	-5.38	> -4.00	> -4.00
**Prostate Cancer**			
PC-3	-4.53	> -4.00	> -4.00
DU-145	> -4.00	> -4.00	> -4.00
**Breast Cancer**			
MCF7	< -8.00	< -8.00	> -4.00
NCI/ADR-RES	> -4.00	> -4.00	> -4.00
MDA-MB-231/ATCC	-4.82	> -4.00	> -4.00
HS 578T	-4.45	> -4.00	> -4.00
MDA-MB-435	> -4.00	> -4.00	> -4.00
BT-549	> -4.00	> -4.00	> -4.00
_MID	-4.87	-4.08	-4.0
Delta	3.13	3.92	0
Range	4.0	4.0	0.0

Axis scale (GI50, TGI, LC50): +3 +2 +1 0 -1 -2 -3
